# Silver ciprofloxacin (CIPAG): a multitargeted metallodrug in the development of breast cancer therapy

**DOI:** 10.1007/s00775-024-02048-y

**Published:** 2024-04-06

**Authors:** Christina N. Banti, Foteini D. Kalousi, Anna-Maria G. Psarra, Eleni E. Moushi, Demetres D. Leonidas, Sotiris K. Hadjikakou

**Affiliations:** 1 Department of Chemistry, University of Ioannina, 45110 Ioannina, Greece; 2 Department of Biochemistry and Biotechnology, University of Thessaly, Larissa, Greece; 3 Department of Life Sciences, The School of Sciences, European University Cyprus, Nicosia, Cyprus; 4 Institute of Materials Science and Computing, University Research Centre of Ioannina (URCI), Ioannina, Greece

**Keywords:** Biological inorganic chemistry, Silver(I) compounds, Ciprofloxacin, Metalloantibiotics, Antitumor activity, Molecular mechanism

## Abstract

The anti-proliferative activity of the known metalloantibiotic {[Ag(CIPH)_2_]NO_3_∙0.75MeOH∙1.2H_2_O} (**CIPAG**) (CIPH = ciprofloxacin) against the human breast adenocarcinoma cancer cells MCF-7 (hormone dependent (HD)) and MDA-MB-231 (hormone independent (HI)) is evaluated. The in vitro toxicity and genotoxicity of the metalloantibiotic were estimated toward fetal lung fibroblast (MRC-5) cells. The molecular mechanism of the **CIPAG** activity against MCF-7 cells was clarified by the (i) cell morphology, (ii) cell cycle arrest, (iii) mitochondrial membrane permeabilization, and (iv) by the assessment of the possible differential effect of **CIPAG** on estrogen receptor alpha (ERα) and estrogen receptor beta (ERβ) transcriptional activation, applying luciferase reporter gene assay. Moreover, the ex vivo mechanism of **CIPAG** was clarified by its binding affinity toward calf thymus (CT-DNA).

**Graphical abstract:**

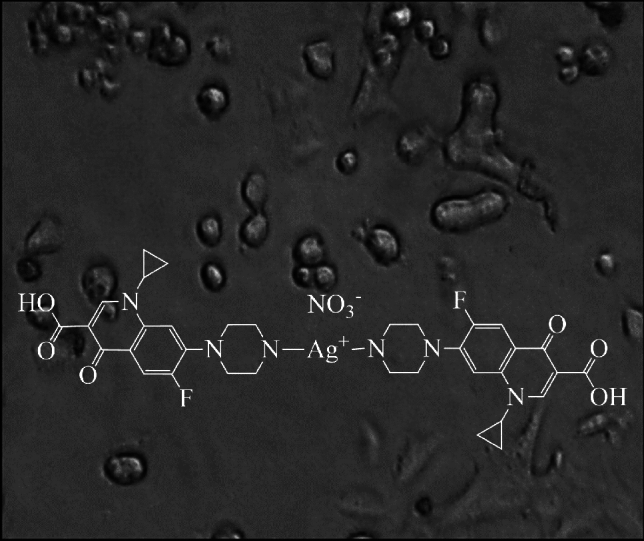

**Supplementary Information:**

The online version contains supplementary material available at 10.1007/s00775-024-02048-y.

## Introduction

Chemotherapy stands out as among the most effective treatments for cancer. Antitumor antibiotics like doxorubicin or daunomycin are widely recognized among clinically chemotherapeutics in use [1]. Nowadays, antibiotics have found application as anticancer medications, notably in cases of cancers related to bacteria, such as gastric and cervical cancers [1]. Thus, antibiotics not only serve as drugs against bacterial infections but also exhibit effectiveness in impeding the growth of cancer cells [2].

Ciprofloxacin, a fluoroquinolone, is widely utilized as a broad-spectrum antibiotic known for its minimal side effects [3]. Fluoroquinolones, a class of antibiotics, function by inhibiting both bacterial DNA gyrase and a type II topoisomerase called topoisomerase IV [1]. Furthermore, apart from possessing antitumor characteristics, fluoroquinolones exhibit diversified biological profiles, showcasing properties that extend to being anti-tubercular, anti-HIV, anti-malarial, anti-Alzheimer, etc. [4]. Especially, ciprofloxacin induces time- and dose-dependent growth inhibition and apoptosis of a plethora of cancer cells, such as prostate, colorectal, leukemia, and breast cell lines [1, 3]. The suggested apoptotic mechanism of ciprofloxacin involves the disruption of mitochondrial membrane potential, followed by a secondary activation of caspase-8 [1]. Moreover, it inhibits cell proliferation by causing damage to mitochondrial DNA and interacts with the mitochondrial topoisomerase II isoform [5].

**Chart 1:**
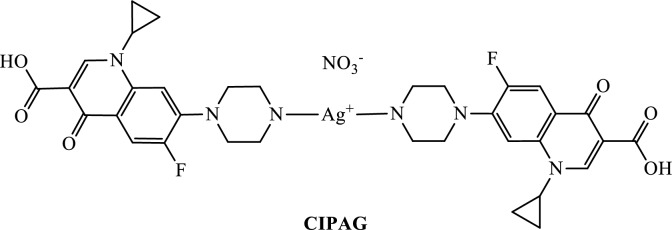
Molecular formula of **CIPAG** [6]

In the course of our studies for the development of new efficient targeted chemotherapeutics [6–12], the in vitro anti-proliferative activity of the conjugate of silver(I) with ciprofloxacin {[Ag(CIPH)_2_]NO_3_∙0.75MeOH∙1.2H_2_O} (**CIPAG**) (CIPH = ciprofloxacin) (Chart 1) against MCF-7 (HD) and MDA-MB-231 (HI) cells, was screened. In vitro assessments were conducted to evaluate the toxicity and genotoxicity on MRC-5 cells. A range of tests was employed to elucidate the molecular mechanism of **CIPAG** against MCF-7 cells, both in vitro and ex vivo. Moreover, **CIPAG** demonstrates significantly greater antimicrobial efficacy (up to 148 times) against bacterial strains *P. aeruginosa*, *S. epidermidis,* and *S. aureus* compared to the commercially accessible hydrochloride salt of ciprofloxacin [6]. The in vivo toxicity of **CIPAG** was assessed using the *Allium cepa* test [6]. Findings indicate that there were no alterations in the mitotic index, even at the tested concentration of 30 μΜ, suggesting the absence of mutagenic or genotoxic effects of **CIPAG** in vivo*.*

## Results and discussion

### General aspects

The metalloantibiotic **CIPAG** was synthesized, following a previously documented method [6]. In a nutshell, a reaction of silver nitrate and ciprofloxacin in a 1:2 molar ratio was processed in a solution of methanol and acetonitrile. Crystals of **CIPAG** were grown by slow evaporation of methanol/acetonitrile solution. The crystal and molecular structures of **CIPAG** and the zwitterionic form of ciprofloxacin were refined using X-ray diffraction data and have been deposited in the Cambridge Crystallographic Database with the CCDC numbers 1537319 and 1,537,320, respectively, while they are detailed described in Ref. 6. A molecular diagram of **CIPAG** is illustrated in Chart 1 [6]. Here, we describe the structure and its charge distribution briefly. **CIPAG** exhibits ionic characteristics. Two neutral ciprofloxacin ligands are coordinated to silver(I) ion via the piperazinic nitrogen atoms [6]. The positive charge located in a silver ion is counterbalanced by a negative charged nitro group, as illustrated in Chart 1 [6]. A different coordination mode, however, is adapted in the case of copper and tin complexes, with ciprofoxacine. In these cases, the ligands are bonded to the metal centers through one keto and one carboxylic oxygen atoms, [13–16]. The coordination arrangement in **CIPAG** entails two nitrogen atoms from the ciprofloxacin ligands binding to the Ag(I) ion, establishing a disorder linear conformation around the silver(I) ion (N1–Ag–N4 = 169.47°). Methanol and water molecules are involved in a network of hydrogen bonding interactions, solvating the crystal structure [6]. The confirmation of **CIPAG’s** formation in solid state was established through attenuated total reflectance–Fourier transform infra-red (ATR–FTIR) and X-ray fluorescence (XRF) spectroscopies [6]. Moreover, its behavior in solution was investigated using ultra-violet (UV) and ^1^H NMR spectroscopies [6]. **CIPAG** exhibits solubility in DMSO, CH_2_Cl_2_, MeOH, and MeCN [6].

The structural integrity of **CIPAG** in various mediums such as DMSO, double-distilled water (ddw), and Dulbecco’s modified Eagle medium (DMEM), validated via UV spectroscopy has been already reported [6]. Figure S1 shows the UV spectra of **CIPAG** in DMSO, ddw, and DMEM over a 48-h period, aligning with the 48-h incubation duration used in the biological experiments.

### X-ray fluorescence spectroscopy (XRF)

**Fig. 1:**
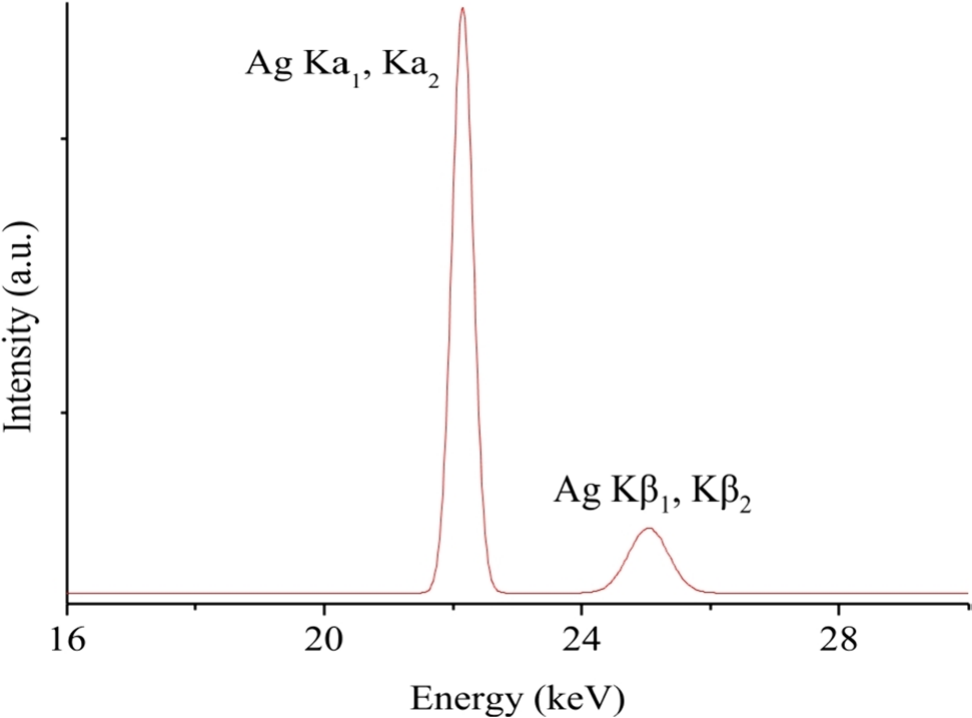
XRF spectrum of **CIPAG**. The Ag Kα peak was used for quantitative determination of Ag in the sample

The XRF spectrum of **CIPAG** powder validates the existence of silver within the complex (Fig. 1). The silver content in **CIPAG** was found to be 12.48% w/w, whereas the calculated silver content for it was 11.81% w/w.

## Biological studies

### Antiproliferative studies

**CIPAG**, alongside ciprofloxacin hydrochloride (**CIPH**_**2**_^**+**^**·Cl**^**−**^), the commercially available antibiotic drug, underwent assessment for their anti-proliferative effects on adenocarcinoma cell lines MCF-7 (hormone-dependent (HD)) and MDA-MB 231 (hormone-independent (HI)) using the sulforhodamine B (SRB) assay. This evaluation was conducted following a 48-h incubation period. The effectiveness of **CIPAG** is quantified in terms of IC_50_ values, representing the concentration causing a 50% inhibition of cell proliferation.

The metalloantibiotic **CIPAG** demonstrates increased efficacy (lower IC_50_ values) against both MCF-7 (7.3 ± 0.4 μΜ) and MDA-MB 231 cells (10.8 ± 0.4 μΜ) in contrast to (**CIPH**_**2**_^**+**^**·Cl**^−^) (IC_50_ values of 417.4 ± 28.2 and 210 ± 4.4 μΜ against MCF-7 and MDA-MB-231 cells, respectively [17]). Therefore, **CIPAG** showcases significantly higher activity, 60-fold against MCF-7 cells and 20-fold against MDA-MB-231 cells, compared to the unbound commercial antibiotic drug (**CIPH**_**2**_^**+**^**·Cl**^**−**^**)**. Furthermore, its higher (~ 30%) anti-proliferative impact on hormone-dependent (MCF-7) compared to hormone-independent (MDA-MB 231) cells suggests the potential involvement of hormone receptors in **CIPAG's** mechanism of action. In comparison to cisplatin, with IC_50_ values of 5.50 ± 0.40 μΜ against MCF-7 and 26.7 ± 1.1 μΜ against MDA-MB-231 [7, 8], **CIPAG** exhibits greater activity than cisplatin by 2.5-fold against MDA-MB-231 cells but lower efficacy by 0.8-fold against MCF-7 cells.

The in vitro toxicity of both **CIPAG** and ciprofloxacin was assessed on normal human fetal lung fibroblast (MRC-5) cells, yielding IC_50_ values of 5.9 ± 0.3 μΜ and > 30 μΜ, respectively. **CIPAG** demonstrates a comparatively lower toxicity profile compared to cisplatin. The therapeutic potency index (TPI) values of **CIPAG** (TPI = [IC_50_(non-cancerous cells)]/[IC_50_(cancerous cells)]) lie at 0.8 for MCF-7 cells and 0.5 for MDA-MB 231 cells. In contrast, the corresponding TPI values for cisplatin are 0.20 for MCF-7 cells and 0.04 for MDA-MB-231 cells [7, 8]. This indicates that **CIPAG** displays reduced toxicity toward non-cancerous cells compared to cisplatin.

### Cell morphology studies

**Fig. 2:**
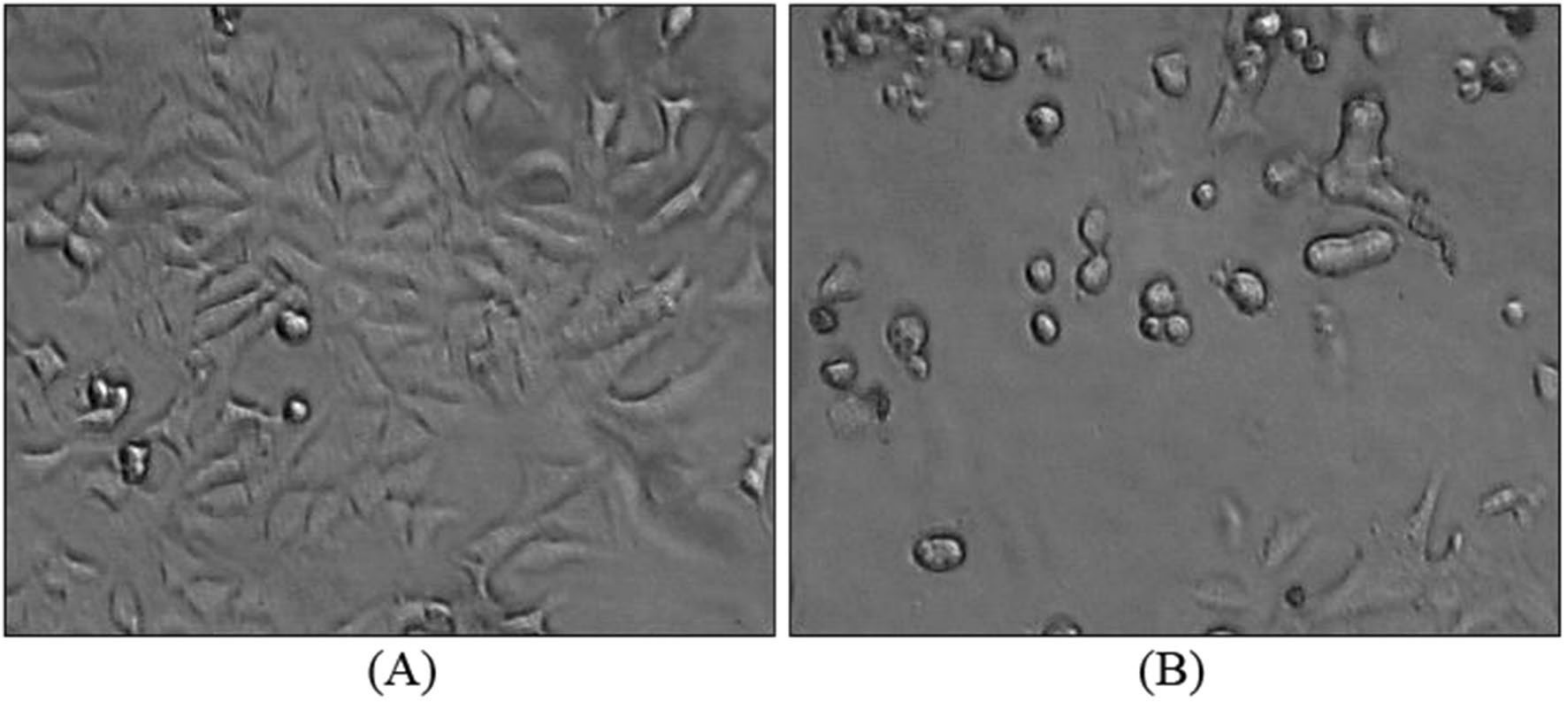
Morphology of the untreated MCF-7 cells (**A**) and their alterations observed when they are treated with **CIPAG** (**B**) at its IC_50_ value for 48 h

To explore the molecular mechanism of **CIPAG**, the alterations in MCF-7 cell morphology were examined via phase-contrast microscopy following a 48-h exposure to its IC_50_ concentration (Fig. 2)*.* Variances in cell morphology were noted between the treated and untreated cells. Specifically, the cells subjected to **CIPAG** treatment exhibited shrinkage, detachment, and a rounded shape [7–10, 18]. Cell shrinkage stands out as one of the most frequently observed morphological changes present in almost every occurrence of apoptotic cells [7–10, 18]. Following cell shrinkage, this tends to lose its contact with adjacent cells and detach from the substrate, leading to the characteristic rounded morphology observed in apoptotic cells [7–10, 18]. These alterations align with the typical morphological features associated with cells undergoing apoptosis, indicating that **CIPAG** might induce programmed cell death in MCF-7 cells.

### Cell cycle arrest

The deregulation of the cell cycle plays a pivotal role in the onset and advancement of cancer, leading to unbridled cell growth and excessive cell proliferation [18]. Studying the cell cycle arrest caused by anticancer agents yields crucial insights into how these agents function and their mechanisms of action against cancer [7, 8]. Furthermore, since apoptotic cells exhibit decreased DNA content (hypodiploid), a distinct sub-G_1_ phase is manifested in the DNA histogram obtained through flow cytometry analysis [19].

**Fig. 3:**
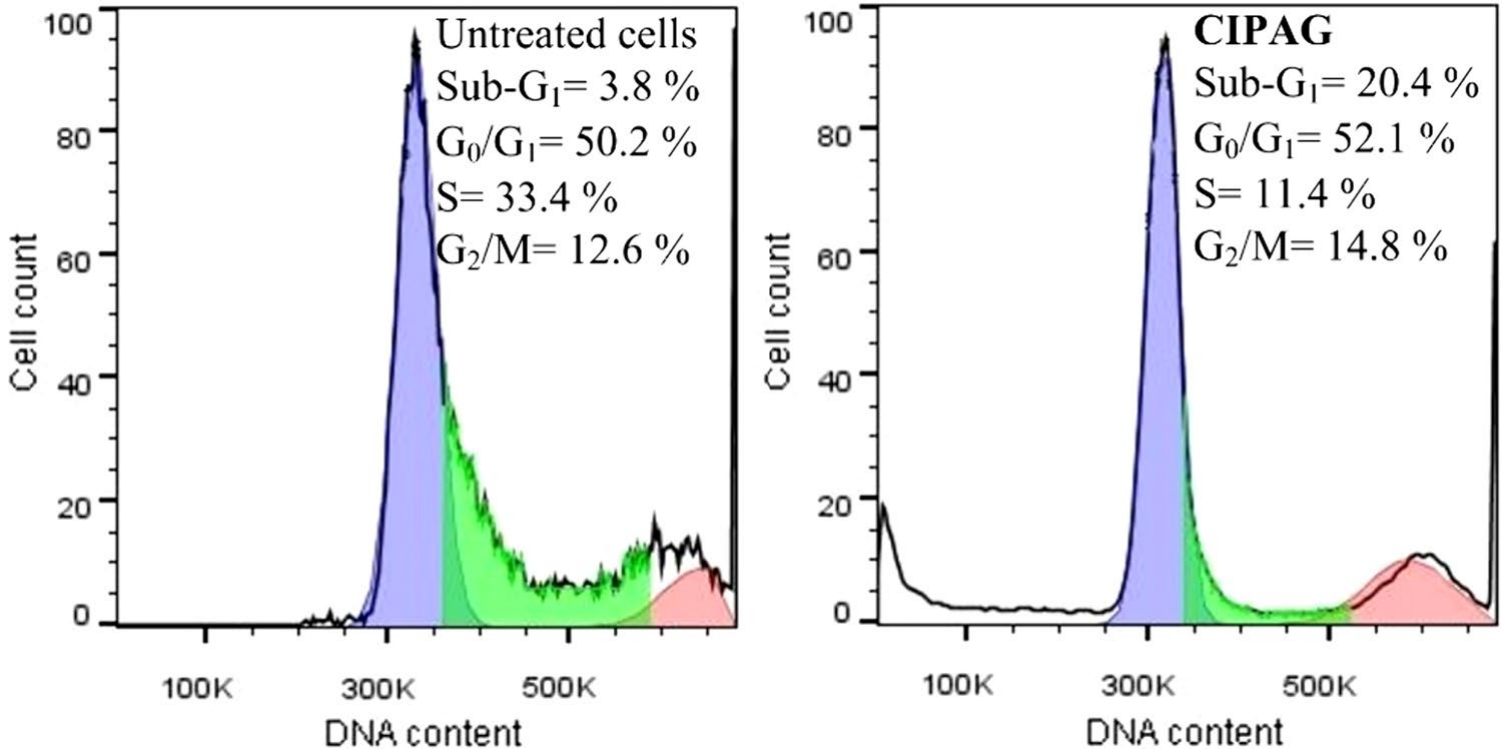
Effect of **CIPAG** on the cell cycle of untreated MCF-7 cells and treated ones. Numbers of cells in the sub-G_1_, G_0_/G_1_, S, and G_2_/M phase are indicated

To explore whether **CIPAG’s** anti-proliferative effects relate to cell cycle arrest, the DNA content of treated and untreated with **CIPAG**, MCF-7 cells was evaluated through flow cytometry analysis. Treatment of MCF-7 cells with **CIPAG** in its IC_50_ value resulted in an increase in the accumulated cells in the G_2_/M phase (14.8% in the case of treated cells instead of 12.6% for untreated cells) and those in G_0_/G_1_ phase (52.1% for the treated cells compared to 50.1% for untreated cells) (Fig. 3). This coincided with a decreasing of the number of cells in the S phase (11.4% for treated cells compared to 33.4% for untreated cells) (Fig. 3). There’s a notable rise in the population of apoptotic cells within the sub-G_1_ phase, reaching 20.4% in cells incubated with **CIPAG** compared to the control group, which stands at 3.8%. Therefore, **CIPAG** suppresses cell proliferation by impeding DNA synthesis, disrupting proper DNA replication, inducing arrest in cell cycle progression within the G_0_/G_1_ and S phases, ultimately leading to apoptosis [7–9, 20].

### Loss of the mitochondrial membrane permeabilization (MPP)

In contemporary metallo-therapeutics, a key objective involves targeting the disruption of mitochondrial membrane permeability within tumor cells [7–9]. Additionally, the decrease in mitochondrial membrane permeabilization (MMP) is recognized as an early event in the apoptotic pathway [21]. Therefore, the impact of **CIPAG** on the mitochondrial membrane function of MCF-7 cells, pre-exposed to it at its IC_50_ value for 48 h, is assessed. This assay relies on the fluorescence of a cationic hydrophobic dye that accumulates in the mitochondria's membrane. When the mitochondrial membrane potential collapses, the release of cytochrome c into the cytosol is followed and the dye is quenched [7–9]. The % fluorescence quenching of treated MCF-7 cells by **CIPAG** is 20.2%. For cisplatin, there's a 54.9% reduction in the emitted radiation from the dye of MCF-7 cells. These experimental results further validate the apoptosis previously inferred through cell morphology and cell cycle arrest.

### Assessment of the possible differential effect of CIPAG on ERα and ERβ transcriptional activation

To evaluate the possible differential effect of **CIPAG** on ERα and ERβ transcriptional activation, estrogen responsive luciferase-based reporter assay was performed in HEK293 cells transiently transfected with a pEGFPC2ERα or a pEGFPC2ERβ construct and subsequently treated with **CIPAG** at concentration of 5 and 10 μΜ for 48 h. Relative luciferase activity revealed no differential effect of **CIPAG** on ERα and ERβ transcriptional activation in the absence or presence of estradiol (Figure S2). This effect may indicate that the **CIPAG** effect on viability of breast cancer cells is estrogen receptors independent. On the other hand, this effect in conjunction with the results from SRB assay, showing differential **CIPAG** action on ERα-positive MCF-7 and almost ERα-negative MDA-MB-231 cells, could possibly indicate that the presence of other regulatory molecules, components of the transcriptional machinery complex, present in ERα-positive MCF-7 or ERα-negative MDA-MB-231 cells, but not in HEK293 cells, play crucial role in the outcome of the **CIPAG** effect on MCF-7 cells viability. Structural conformational changes of ERα and ERβ that can be induced via their interaction with other regulatory molecules in breast cancer cells, may affect estrogens receptors’ binding or interaction with ERs potential agonists or antagonists, including metalloestrogens as has been previously observed [22, 23]. Alternatively, **CIPAG** possible direct or indirect interference with ERs may induce conformational changes to ERs that modulate receptors’ binding or recruitment of other regulatory molecules of the transcriptional machinery crucial for ERs transcriptional activation [24].

### Ex vivo mechanism

The ex vivo mechanism of **CIPAG** was elucidated through its binding affinity for calf thymus DNA (CT-DNA).

#### DNA-binding studies

Metallodrugs are recognized for their ability to bind to DNA, consequently disrupting its function [25]. Thus, the DNA-binding mode of **CIPAG** has been investigated using various methods including viscosity measurements, absorption titration, fluorescence spectroscopy, and DNA thermal denaturation studies.

#### Viscosity studies

Viscosity measurement is a sensitive technique shedding light on the various modes of DNA binding. Therefore, when an agent interacts with CT-DNA in an intercalation mode, like ethidium bromide with DNA, the double helix unravels and stretches, causing a rise in relative viscosity [26]. For groove binding or electrostatic modes, there is no observed impact on DNA length and no notable change in viscosity [27]. When an agent induces DNA strand cleavage, both the DNA length and viscosity notably decrease [26, 27]. On the other hand, when an agent like cisplatin forms a covalent bond with DNA, the solution viscosity decreases due to kinking in the DNA backbone and a shortened axis length of the DNA helix [26, 27].

**Fig. 4:**
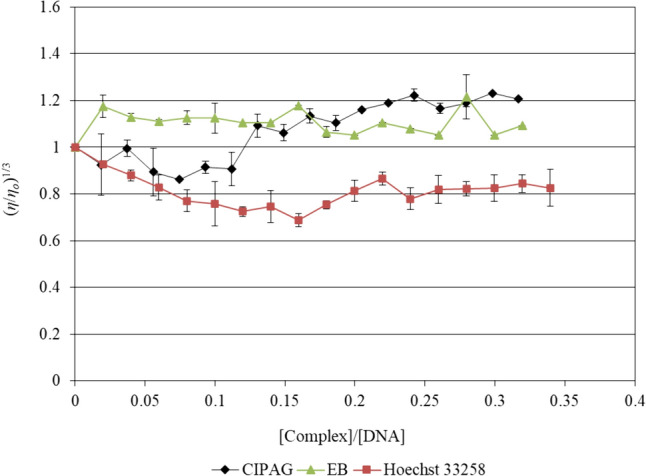
Relative viscosity of CT-DNA with increasing concentrations of **CIPAG**, Hoechst 33,258 and Ethidium Bromide ([DNA] = 10 mM, *r* = [compound]/[DNA], *η* is the viscosity of DNA in the presence of the compounds and *η*_*o*_ is the viscosity of DNA alone)

The values of relative specific viscosity (*η/η*_*ο*_)^1/3^ were plotted vs the values *r* = [complex]/[DNA] (Fig. 4). For **CIPAG**, there is no notable alteration in viscosity observed, suggesting a groove binding mode or electrostatic interaction, akin to the minor groove binding agent Hoechst 33,258 (Fig. 4).

#### UV–Vis spectroscopic study

Electronic spectroscopy is a valuable tool in investigating the interaction between a DNA binder and DNA. The nature of the interaction between the binder and DNA can be assessed by monitoring the changes in absorption spectrum as the DNA solution undergoes titration with increasing concentrations of the binder. Therefore, (i) a *hypochromic* effect, i.e., absorbance decreasing at the *λ*_max_ of CT-DNA (260 nm) with a subsequent significant shift (> 15 nm), either toward *bathochromic* (red) or *hypsochromic* (blue) shifts, in the *λ*_max_ (260 nm) of CT-DNA indicates an intercalation interaction between the binder and CT-DNA. (ii) a weak *hyperchromic* effect, i.e., absorbance increasing at the *λ*_max_ of CT-DNA, or weak *hypochromism*, with subsequent negligible or no *bathochromic* or *hypsochromic* shifts in the *λ*_max_ of CT-DNA, suggests electrostatic interactions or weak interaction. However, in the event of a strong *hyperchromic* change (> 40%) in the absorbance of CT-DNA, with no shifts in the *λ*_max_, indicate denaturation of the hydrogen bonds and destruction of the secondary DNA conformation. Finally, (iii) groove binding with hydrogen bonds or van der Waals interactions is concluded upon *hyperchromic* change in the absorbance at the *λ*_max_ of CT-DNA, with subsequent small (< 8 nm) *bathochromic* or *hypochromic* shift in the *λ*_max_ of CT-DNA [7, 9–11, 28].

Figure S3 illustrates the UV–Vis spectra of a CT-DNA solution both in the absence and presence of **CIPAG** at different r values (*r* = [agent]/[DNA]) while maintaining a constant [DNA]. An observed hyperchromic effect of up to 19.4% (Figure S3), suggests potential groove binding or damage to the secondary structure of DNA [8–10, 28]. Furthermore, it is acknowledged that groove binders or intercalators feature aromatic ring systems like **CIPAG**, enabling them to establish π···π stacking interactions with DNA bases, thereby aiding their attachment within DNA grooves [28]. The binding constant (*K*_*b*_) of **CIPAG** was derived from the slope and y-intercept of the graph correlating [DNA]/(*ε*_a_ – *ε*_f_) against [DNA], utilizing the Wolfe–Shimmer equation (Figure S4). The calculated *K*_*b*_ value was determined (5.8 ± 1.4) × 10^4^ M^−1^.

#### Fluorescence spectroscopic studies

**Fig. 5:**
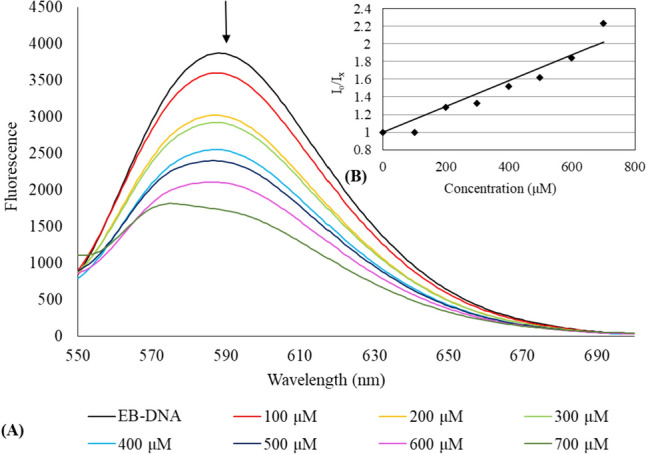
**A** The emission spectrum of the CT-DNA–EB complex excited at *λ*_exc_ = 527 nm in the presence of **CIPAG** ([EB] = 2.3 μM, [DNA] = 26 μM, [complex] = 0–700 μM). The arrow indicates the change in intensity with increasing complex concentration. The inset **B** displays plots of emission intensity *I*_o_/*I*_x_ against [agent]

Further investigation into the binding properties of **CIPAG** with CT-DNA was conducted using fluorescence spectroscopy. Ethidium bromide (EB), known for its strong intercalation between DNA base pairs, emits intense fluorescent light in the presence of DNA. The expulsion of EB from the DNA–EB complex due to a metallodrug, resulting in the quenching of emitted light, signifies an intercalative or minor groove binding mode [8–10, 26, 27]. The emission spectra of CT-DNA–EB, in the absence and presence of **CIPAG**, when excited at *λ*_exc_ = 527 nm, are depicted in Fig. 5.

The emitted light at 588 nm from the CT-DNA–EB complex undergoes a 55.2% reduction in fluorescence intensity compared to the initial fluorescence intensity of the EB-DNA solution. The value of apparent binding constant (*K*_app_) was calculated and the concentration of the drug at 50% reduction of the fluorescence is derived from the diagram of (*I*_*x*_*/I*_0_) (*I*_*0*_ and *I*_*x*_ are the emission intensities of the CT-DNA–EB in the absence and presence of metallodrug) vs the concentration of **CIPAG** (Fig. 5) [8–10, 26, 27]. The calculated *K*_app_ value fell within the range of 10^4^–10^5^ M^−1^ ((3.9 ± 0.9) × 10^4^ M^−1^), indicating a minor groove binding tendency [8–10].

#### DNA thermal denaturation study

DNA melting studies can give evidence about the binding mode and the strength of the drug-DNA interaction. On increasing temperature of DNA solution, the bases stacking interactions and hydrogen bonds between the bases of the double-stranded DNA are disturbed, resulting in a hyperchromic effect in the absorption spectra (*λ*_max_ = 260 nm), and a ‘sigmoid’ melting transition curve is produced. The DNA melting temperature (*T*_m_) is the temperature at which half of the double-stranded DNA separates into two single strands [29–31]. The *T*_m_ value of CT-DNA is obtained from the transition midpoint of the melting curves based on the relative absorbance (*f*_ss_) *vs* temperature (*f*_ss_ = (*A* – *A*_min_)/(*A*_max_ = *A*_min_), where *A*_min_ is the minimal absorbance of the solution of CT-DNA–drug, at a temperature T, A is the absorbance of CT-DNA–drug corresponding to a specific temperature and A_max_ is the maximum absorbance of CT-DNA–drug, respectively [30, 31].

**Fig. 6:**
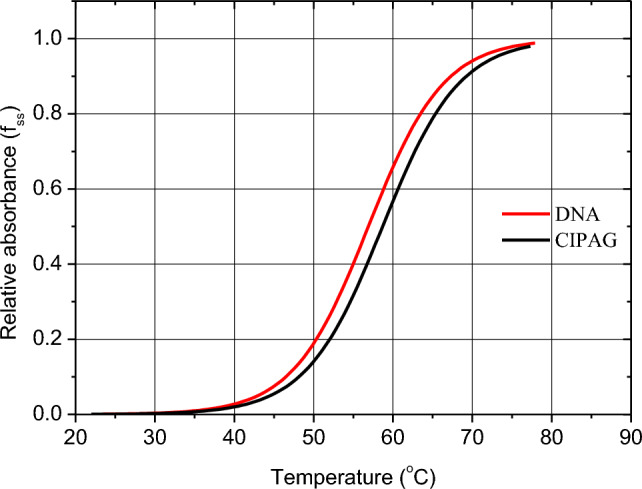
Melting curves of CT-DNA in the absence and presence of **CIPAG**, CT-DNA = 2.0 × 10^–5^ M; *C*_drug_ = 10^–6^ M in 1 mM trisodium citrate, 10 mM NaCl (pH = 7.0)

Figure 6 shows the DNA thermal denaturation curves in the absence and in the presence of **CIPAG**. The *T*_m_ value for free CT-DNA is 59.5 ± 1.0 °C, while in the presence of **CIPAG** is 60.6 ± 1.3 °C. Since, the Δ*T*_m_ value is lower than 2 °C, a groove binding or electrostatic interaction mode is concluded, which is in accordance with the previous studies.

## Conclusions

Antibiotics, like ciprofloxacin, are recognized for their potential use as anticancer treatments due to their ability to trigger apoptosis. In our earlier research, we synthesized, characterized, and examined the antibacterial activity of the silver(I) conjugate with ciprofloxacin, denoted as {[Ag(CIPH)_2_]NO_3_∙0.75MeOH∙1.2H_2_O} (**CIPAG**). Nevertheless, the absence of in vitro or in vivo genotoxicity of **CIPAG**, even up to the tested concentration of 30 μΜ, prompts us to investigate its anti-proliferative mechanism against breast cancer cells.

The metalloantibiotic **CIPAG** demonstrates notably higher cytotoxic compared to free ciprofloxacin activity (60-fold higher against MCF-7 cells and 20-fold against MDA-MB-231 cells). However, based on the relative luciferase activity, no discernible variation in the effect of **CIPAG** on ERα and ERβ transcriptional activation was observed in HEK293 cells, regardless of the absence or presence of estrogens. Additionally, **CIPAG** demonstrates a 2.5-fold higher activity than cisplatin against MDA-MB-231 cells and a slightly lower efficacy by 0.8-fold against MCF-7 cells. However, the TPI (therapeutic index) values of **CIPAG** are more favorable compared to those of cisplatin, suggesting lower in vitro toxicity toward non-cancerous cell lines.

The apoptotic mechanism of **CIPAG** was confirmed through observed changes in the cell morphology of treated MCF-7 cells and an increase in the percentage of cells in the sub-G_1_ phase. The cell cycle analysis further indicates that **CIPAG** induces arrest in the progression of the cell cycle at both the G_0_/G_1_ and S phases. This suggests its role in inhibiting cell proliferation by impeding DNA synthesis and disrupting the proper replication of DNA. The ex vivo studies suggest a potential groove binding or electrostatic interaction mode of **CIPAG** with *K*_b_ or *K*_app_ within the range of 10^4^ M^−1^.

## Experimental

### Materials and methods

Ciprofloxacin hydrochloric salt was offered from Help Pharmaceutical. Reagent-grade solvents (Merck) were employed without additional purification. Dimethyl sulfoxide and boric acid were from Riedel–de Haen. Dulbecco’s modified Eagle’s medium, (DMEM), fetal bovine serum, glutamine, and trypsin were purchased from Gibco, Glasgow, UK. Phosphate buffer saline (PBS) was purchased from Sigma-Aldrich. Dimethyl sulfoxide and boric acid were from Riedel–de Haen. Calf thymus (CT)-DNA, ethidium bromide (EB), and RNase A were purchased from Sigma-Aldrich were procured from Sigma-Aldrich. The MCF-7, MDA-MB-231, and MRC-5 cell lines used in this study were obtained from ATCC (American Type Culture Collection). Melting point measurements were performed in open tubes using a Stuart Scientific apparatus and remain uncorrected. Elemental analyses for carbon and hydrogen were conducted using the Carlo Erba EA MODEL 1108 elemental analyzer. Infrared spectra spanning from 4000 to 370 cm^−1^ were obtained using a Cary 670 FTIR spectrometer from Agilent Technologies. The ^1^H NMR spectra were recorded using a Bruker AC 250 MHz FT-NMR instrument in DMSO-*d*_*6*_ solution, with chemical shifts (δ) using (CH_3_)_4_Si as an internal reference. Electronic absorption spectra were recorded using a VWR UV-1600 PC series spectrophotometer. Fluorescence spectra were captured utilizing a Jasco FP-8200 Fluorescence Spectrometer. XRF measurements were carried out employing a Rigaku NEX QC EDXRF analyzer situated in Austin, TX, USA.

### Synthesis and crystallization of CIPAG

This was performed as previously descripted [6]. Briefly, a clear solution of 0.5 mmol hydrochloric salt of ciprofloxacin (0.385 g) in water (8 cm^3^) was treated with 0.5 cm^3^ KOH 1 N and the resulting precipitation was filtered off and dried. The white powder was then dissolved in 10 mL methanol / 10 mL acenonitrile and 1 mmol silver nitrate (0.170 g) was added. The mixture was stirred for 30 min and the yellowish solution was kept in darkness. Pale yellow crystals of pure **CIPAG** were grown from slow evaporation of the solution after 2 days. The physicochemical properties (color, melting point, analytical data) as well as the spectroscopic characteristics are identical to those reported [6].

### Biological tests

#### Solvents used

Stock solutions of **1** (0.001 M) in DMSO were freshly prepared and diluted with cell culture media to suitable concentration for the biological experiments including assessment of viability with SRB assay, cell morphology studies, cell cycle arrest and permeabilization of the mitochondrial membrane test. For CT-DNA-binding studies, the experiments were carried out in DMSO/buffer solutions. The biological experiments were replicated three independent times at least.

#### Cell culture

The MCF-7, MDA-MB 231, MRC-5, and HEK293 cell were cultured at 37 °C and 5% CO_2_ humidity, in DMEM, supplemented with 10% FBS, 2 mM L-glutamine and 100 units/ml penicillin/streptomycin. The human embryonic kidney HEK293 cells are characterized by high efficiency in transfections experiments [32].

#### SRB assay

Stock solutions of **1** (0.001 M) were dissolved in DMSO and diluted in cell culture medium to the suitable concentrations (4–30 μΜ). The MCF-7, MDA-MB 231 and MRC-5 cells were plated in 96-well flat-bottom microplates at various cell inoculation densities 6000, 8000, and 2000 cells/well, correspondingly. These cells were incubated for 24 h at 37 °C and they were exposed for 48 h. The evaluation of the cytotoxicity of the compound was determined by means of SRB colorimetric assay at *λ* = 568 nm by giving the percent of the survival cells against the control ones (untreated cells). The SRB assay was carried out as previously described [7–10, 31].

#### Cell morphology studies

MCF-7 cells morphology was observed under an inverse microscope, after incubation of MCF-7 cells by **CIPAG**, for 48 h [7–10, 31].

#### Cell cycle arrest

The MCF-7 cells were seeded into a 24-well plate (70,000 cells per well) and cultured at 37 °C for 24 h. Afterward, the cells were treated with **CIPAG** at its IC_50_ value for 48 h. This assay was performed as reported previously [31].

#### Permeabilization of the mitochondrial membrane test

The MMP assay was performed using the kit which was purchased from sigma Aldrich “Mitochondria Membrane Potential Kit for Microplate Readers, MAK147”. MCF-7 cells were treated with **1** at its IC_50_ value. The fluorescence intensity is measured at *λ*_ex_ = 540 and *λ*_em_ = 590 nm. The experimental outputs include only intensities of fluorescence of the solutions (2 solutions of treated cells in wells and 2 solutions of untreated cells in wells) [7, 8, 31].

#### Estrogen receptor transcriptional activity

For the evaluation of the effect of **CIPAG** on ERalpha (ΕRα) and ERbeta (ERβ) transcriptional activity, ERE-dependent luciferase reporter gene assay was performed in HEK293 cells as previously described [33]. HEK293 cells were selected due to their high efficiency in transfections experiments and due to their low expression levels of estrogen receptors. Transfection of HEK293 cells with either a pEGFC2ERα or a pEGFPC2ERβ construct allowed us to evaluate the possible differential effect of **CIPAG** on ERα and ERβ receptor. Thus, for luciferase assay, 5 × 10^4^ cells were grown on 24-well plates and co-transfected with either an Estrogen responsive luciferase reporter (ERE-Luc) construct, a β-galactosidase reporter construct, and with either a pEGFPC2ERα or a pEGFPC2ERβ construct, for ERα or ERβ activity assessment, respectively. Then, cells were treated, with the indicated amounts of **CIPAG**, in the presence or absence of 10^–9^ M E2. After 6-h incubation, cells were washed in PBSX1, and then lysed in reporter lysis buffer (Promega). The assessment of the activity of the expressed luciferase and β-galactosidase activity was followed. The light emission was measured by a chemiluminometer (LB 9508, www.berthhold.com) and the relative luciferase activity was expressed as normalized luciferase activity against β-galactosidase activity.

#### DNA-binding studies using Uv–vis, fluorescence studies

These studies were performed as previously reported [7–10, 31].

#### Viscosity measurements

This study was carried out as previously reported [7–10, 31]. The kinematic viscosity of DNA solutions with or without **CIPAG** ([**CIPAG**]/[DNA] molar ratios of 0–0.35) was measured by an Ostwald-type viscometer.

#### DNA thermal denaturation study

Thermal melting curves were measured using a Uv–Vis spectrophotometer. The ratio of DNA to metallodrug was 20:1 molar ratio. Thermal melting curves for DNA were determined by following the absorption change at 258 nm in buffer containing 1 mM trisodium citrate, 10 mM NaCl (pH = 7.0) in the absence or presence of **CIPAG** as a function of temperature. The absorbance scale was normalized. The *T*_m_ value of CT-DNA was obtained from the transition midpoint of the melting curves based on the relative absorbance (*f*_ss_) vs temperature (*f*_ss_ = (*A* – *A*_min_)/(*A*_max_ – *A*_min_), where *A*_min_ is the minimal absorbance of the solution of CT-DNA–drug, at a temperature *T*, *A* is the absorbance of CT-DNA–drug corresponding to a specific temperature and *A*_max_ is the maximum absorbance of CT-DNA–drug, respectively. *T*_m_ were determined by applying a Gauss fit to the first derivative of the respective melting profile [31]. The *T*_m_ value was taken as the midpoints of the transition curves, as determined from the maximum of the first derivative. Δ*T*_m_ value were calculated subtracting *T*_m_ for the free nucleic acid from *T*_m_ for each agent. Every reported Δ*T*_m_ value was the average of at least three measurements [31].

### Supplementary Information

Below is the link to the electronic supplementary material.

Supplementary file1 (PDF 367 KB)

## Data Availability

The data included in this article are electronically available by the corresponding authors upon request.
