# Supplementary material for: Silver ciprofloxacin (CIPAG): a multitargeted metallodrug in the development of breast cancer therapy
Source: J Biol Inorg Chem. 2024 Apr 6;29(2):177–86. doi: 10.1007/s00775-024-02048-y (PMC11098868; doi:10.1007/s00775-024-02048-y)
Supplement: Supplementary file 1 — Supplementary file1 (PDF 367 KB) [file 775_2024_2048_MOESM1_ESM.pdf]

Supplementary Information

Silver ciprofloxacin (CIPAG): A multitargeted metallodrug in the development of breast cancer therapy

Chistina N. Banti*[a], Foteini D. Kalousi[b], Anna-Maria G. Psarra[b], Eleni E.Moushi[c], Demetres D. Leonidas[b], Sotiris K. Hadjikakou*[a,d]

[a] Department of Chemistry, University of Ioannina, 45110 Ioannina, Greece.

[b] Department of Biochemistry and Biotechnology, University of Thessaly, Larissa, Greece

[c] Department of Life Sciences, The School of Sciences, European University Cyprus, Cyprus

[d] University Research Center of Ioannina (URCI), Institute of Materials Science and Computing, Ioannina, Greece

*All correspondence should be addressed to:

Dr C.N. Banti (Adjunct Lecturer); email: cbanti@uoi.gr

Dr S.K. Hadjikakou (Professor): e-mail: shadjika@uoi.gr; tel. x30-26510-08374

fax +30-26510-08786

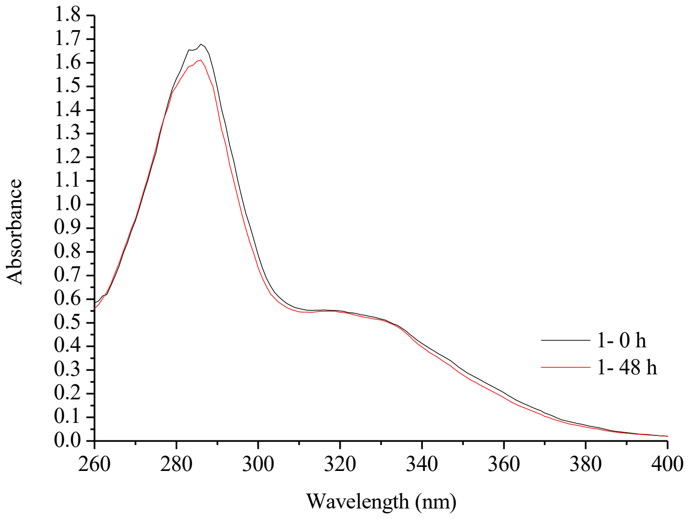

(A)

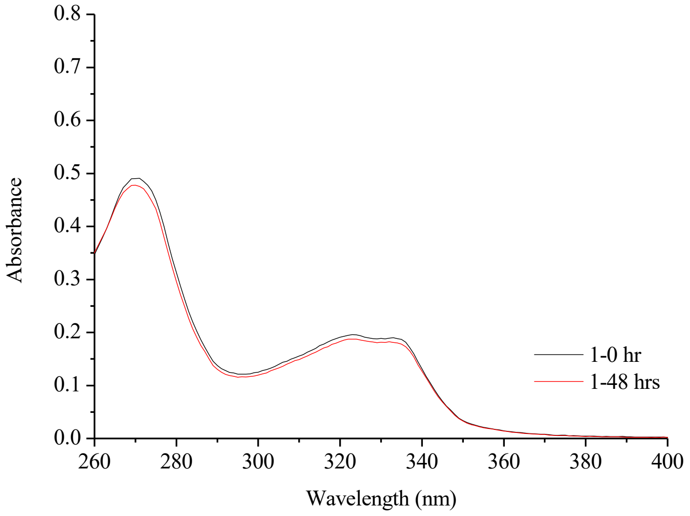

(B)

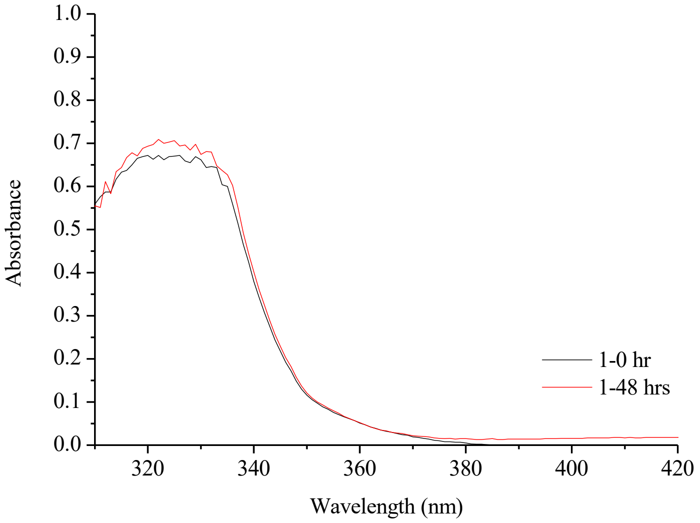

(C)

Figure S1. Initial UV spectra of **CIPAG** in DMSO **(**6.7 10^{-6} M) (**A**), in ddw (10×10^{-6} M) (B) and in DMEM (25×10^{-6}) and the corresponding ones after 48 hr.

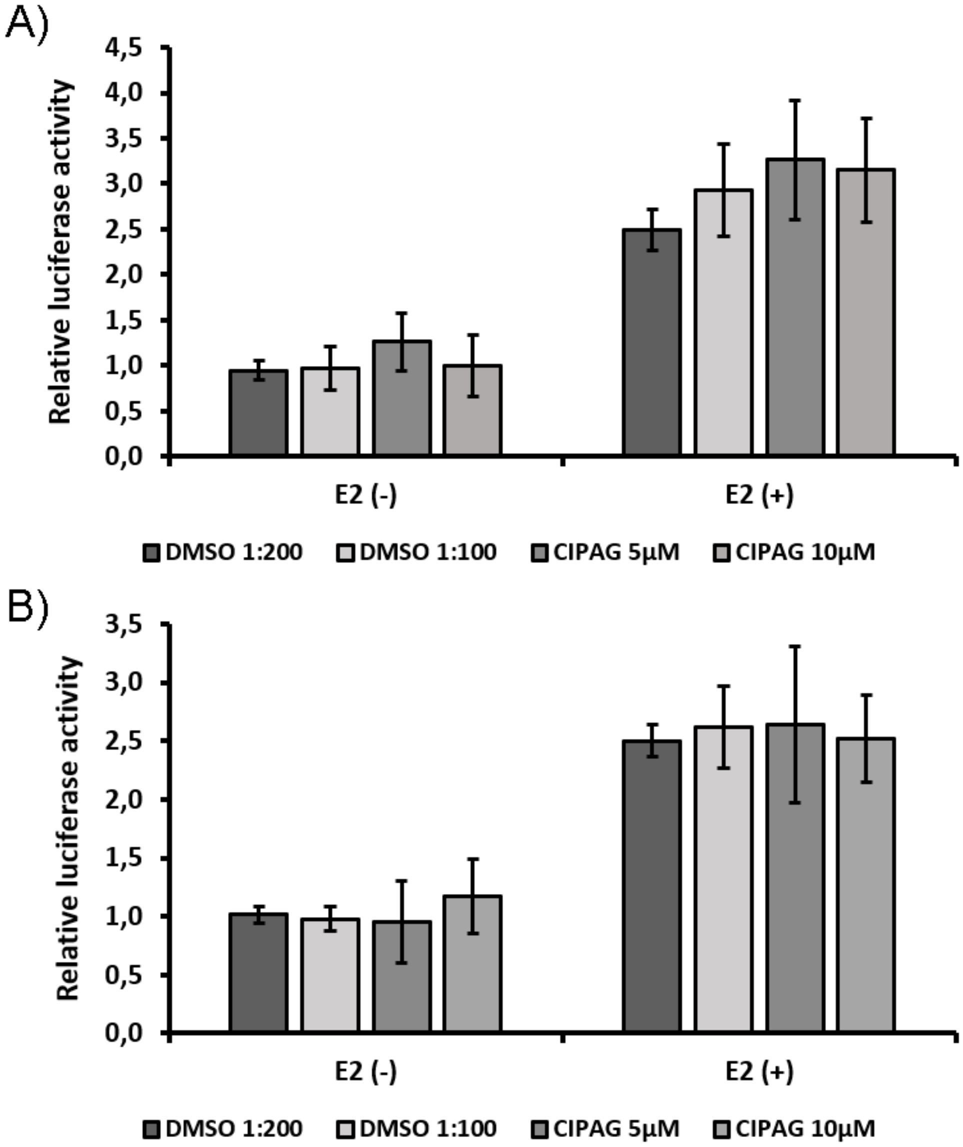

Figure S2. Assesment of the potential differential effect of **CIPAG** on ERα (A) and ERβ (B) transcriptional activation in HEK293 cells. Estrogen dependent Luciferase reporter assay was applied in HEK293 cells, transiently transfected to express ERα or ERβ fused with the GFP protein and subsequently treated with **CIPAG** at concentrations of 5 μM and 10 μM, in the absence or presence of 10^{-9} M E2 for 6 hrs. Control cells were treated with DMSO (1:200 or 1:100) and EtOH (1:1000). Relative luciferase activity was expressed as normalized luciferase activity against β-galactosidase activity. Relative luciferase activity of control cells was set as 1. Data are expressed as the mean ± SD, (n = 3). No statistically significant changes were observed.

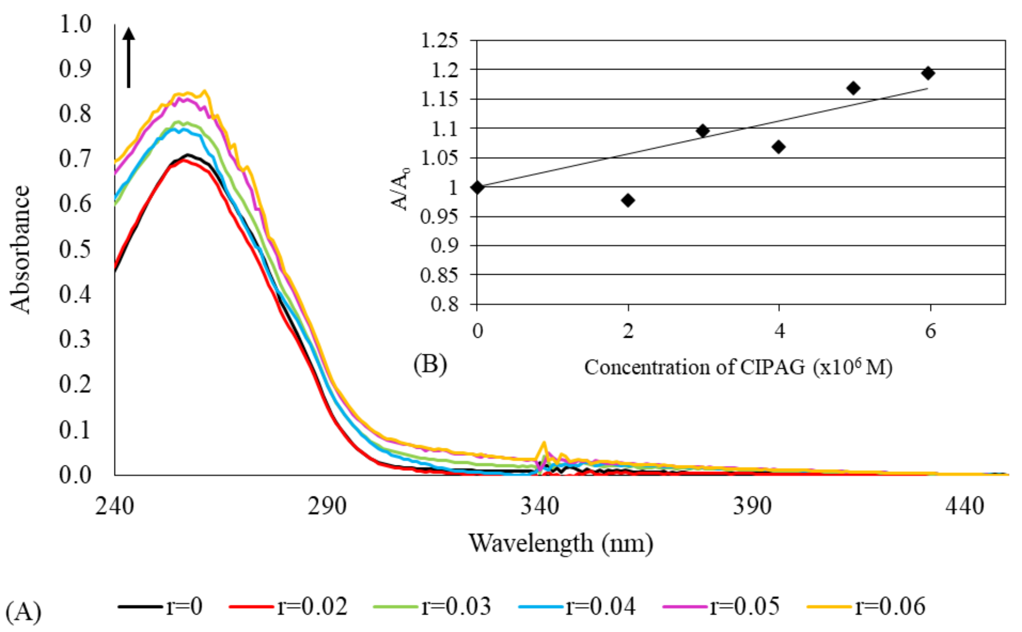

Figure S3. UV spectra of CT DNA in buffer solution in the absence or presence of **CIPAG** at r values of 0-0.06 (r= [complex]/[DNA], [DNA]= 10^{-4} M) and (B) plot of A/Ao vs. [complex] at λ_{max}= 258 nm.

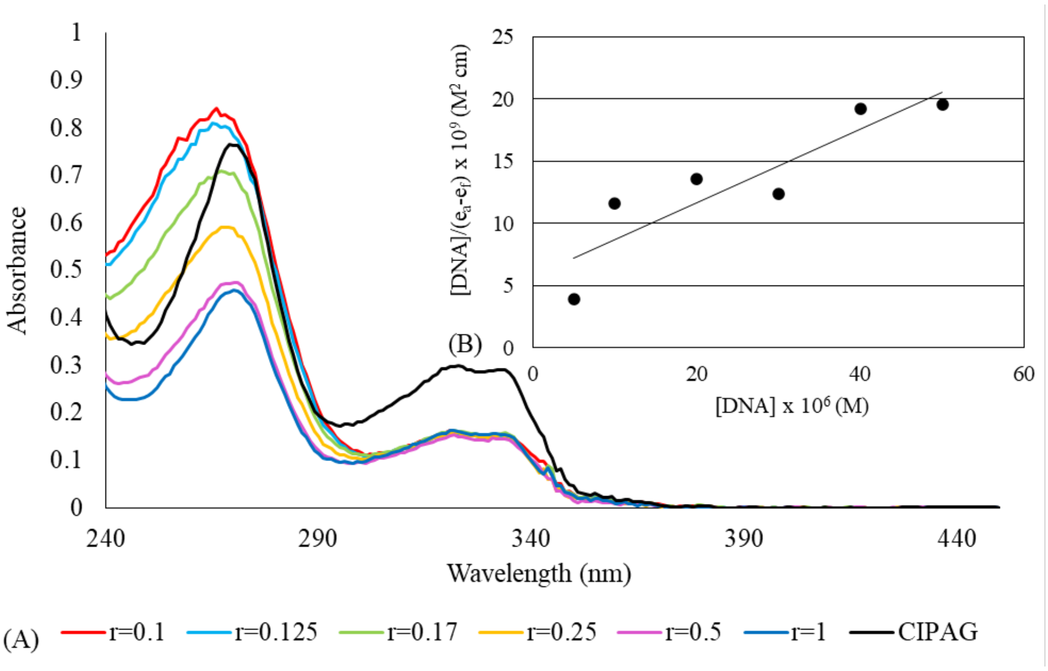

Figure S4. (A) UV spectra of **CIPAG** in the absence or presence of CT DNA at r values of 1, 0.5, 0.25, 0.17, 0.125 and 0.1 (r= [complex]/[DNA], [complex]= 5 μM, [CT-DNA]= 0.005-0.05 mM). (B) Graphical plot of [DNA]/(e_a-e_f) *vs.* [DNA]. (e_a=A_{obsd}/[compound], e_f= the extinction coefficient for the free compound)
